# Correction: Epidemiological study of cervical cord compression and its clinical symptoms in community-dwelling residents

**DOI:** 10.1371/journal.pone.0349334

**Published:** 2026-05-12

**Authors:** Toru Hirai, Koji Otani, Miho Sekiguchi, Shin-ichi Kikuchi, Shin-ichi Konno

In the Materials and Methods section, there is an error in the first sentence. The correct sentence is: This study was approved by the ethics committee of Fukushima Medical University (No. 396).
