# Supplementary material for: Epidemiological study of cervical cord compression and its clinical symptoms in community-dwelling residents
Source: PLoS One. 2021 Aug 27;16(8):e0256732. doi: 10.1371/journal.pone.0256732 (PMC8396744; doi:10.1371/journal.pone.0256732)
Supplement: S1 Table — (DOCX) [file pone.0256732.s001.docx]

**Detail of MRI.**

| Manufacturer | Hitachi | Toshiba |
| --- | --- | --- |
| Product name | AIRIS mate | EXCELART Pianissimo |
| Tesla | 0.2T | 1.0T |
| Slice thickness (mm) | 5 | 4 |
| Slice gap (mm) | 1 | 0.8 |
| Imaging protocol | Turbo spin-echo pulse sequence | |
| TE (ms) | 125 | 110 |
| TR (ms) | 3000 | 3300 |
| No. of participants | 224 | 308 |
