# Supplementary material for: Epidemiological study of cervical cord compression and its clinical symptoms in community-dwelling residents
Source: PLoS One. 2021 Aug 27;16(8):e0256732. doi: 10.1371/journal.pone.0256732 (PMC8396744; doi:10.1371/journal.pone.0256732)
Supplement: S1 File — (DOC) [file pone.0256732.s002.doc]

**name　　　　　　　　M／F　　age　　　　　image number**

**individual code　　　　　　　　　　　　　　　area number**

**doctor interview sheet**

**interviewer**

**complications of internal medicine**

**diabetes**（　no　／　only dietic treatment　／　medication　／　with insulin　／　untreated　）

**hypertonia**　（　no　／　only dietic treatment　／　medication　／　untreated　）

**hyperlipidemia**　（　no　／　only dietic treatment　／　medication　／　untreated　）

**others　：**

**Hospital**　（　Minami aizu hospital　／Tateiwa hoshi medical office／Manaki clinic ）

　　　　　Other hospital：　　　　　　　　　　　　　　　　　　　　　　　　　　）

**Subjective symptoms**

**neck pain**　　　　　　（no　／right　／left　／middle　／both sides）

**stiff shoulder**　　　（no　／right　／left　／middle　／both sides）

**right upper extremity pain**（no　／shoulder　／upper arm　／forearm　／hand　／finger）

**right upper extremity numbness**（no　／shoulder　／upper arm　／forearm　／hand　／finger）

**left upper extremity pain**（no　／shoulder　／upper arm　／forearm　／hand　／finger）

**left upper extremity numbness**（no　／shoulder　／upper arm　／forearm　／hand　／finger）

**body and lower extremity symptoms**（　no　／yes　：location　　　　　　　　　　　　　　　　　　　）

**fall down in the past 1 year**（　no　／one time　／two times　／more than three times　　）

**clumsy hand　no**　／**yes**

　　　　　　　　　　　chopstick：　possible　／awkward　／　only large one　／impossible

　　　　　　　　　　　writing：　possible　／awkward　／　　already impossible　　　／impossible

　　　　　　　　　　fastening buttons：　possible　／　buttons on sleeves　／　big buttons　／impossible

**gait disturbance**　nomal　／capable of fast walking but clumsy

／walks independently when going upstairs but needs support when going downstairs

／walks independently on a level but needs support on stairs

／Able to walk without a support but with a clumsy gait

／Unable to walk on a level without a cane or other support

　　　 ／Able to stand up but unable to walk

／Unable to stand up and walk by any means

**lower extremity arthralgia**　no　／yes　：　hip　、knee、ankle

**operation of lower extremity joint**no　／yes　：　hip　、knee、ankle

　　　　　　　　　　　　　　　　　detail of the operation

**possibility of cervical diseases based on these interviews**

　　　　no　／yes　（　cervical spondylosis　／cervical myelopathy　／cervical radiculopathy　）

**numbness from thumb to middle (ring) finger**　　no　／right　／left　／both hand

**nocturnal awakening due to numbness** no　／yes

**the numbness is relieved by swing hand** no　／yes

**possibility of carpal tunnel syndrome based on these interviews**

no ／yes

**numbness of little (ring) finger**no　／right　／left　／both hand

**when the elbow is bended, the numbness appears**no　／yes

**possibility of cubital tunnel syndrome based on these interviews**no　／yes

**medical examination or treatment history of orthopedic**
