# Supplementary material for: Epidemiological study of cervical cord compression and its clinical symptoms in community-dwelling residents
Source: PLoS One. 2021 Aug 27;16(8):e0256732. doi: 10.1371/journal.pone.0256732 (PMC8396744; doi:10.1371/journal.pone.0256732)
Supplement: S2 File — (DOC) [file pone.0256732.s003.doc]

**氏名　　　　　　　　男／女　　年齢　　　　　MR / レ線番号**

**検診個人コード　　　　　　　　　　　　　　　地区番号**

**医師用問診表**

**問診者　　　　他：**

**内科併存症**

**糖尿病**（　なし　／　食事のみ　／　経口薬　／　インスリン　／　未治療　）

**高血圧**　（　なし　／　食事のみ　／　経口薬　／　未治療　）

**高脂血症**　（　なし　／　食事のみ　／　経口薬　／　未治療　）

**その他　：**

**治療先**　（　南会津病院　／　舘岩星医院　／　まなき診療所　／

　　　　　他（市町村名と医院名）：　　　　　　　　　　　　　　　　　　　　　　　　　　）

**自覚症状**

**頚部痛**　　　　　　（なし　／　右　／　左　／　中央　／　両側）

**肩こり**　　　　　（なし　／　右　／　左　／　中央　／　両側）

**右上肢痛**　　　　　（　なし　／　肩　／　上腕　／　前腕　／　手　／　指　）

**右上肢しびれ**　　　（　なし　／　肩　／　上腕　／　前腕　／　手　／　指　）

**左上肢痛**　　　　　（　なし　／　肩　／　上腕　／　前腕　／　手　／　指　）

**左上肢しびれ**　　　（　なし　／　肩　／　上腕　／　前腕　／　手　／　指　）

**体幹や下肢症状**（　なし　／　あり　：部位　　　　　　　　　　　　　　　　　　　）

**過去1年の転倒の有無**（　なし　／　1回　／　2回　／　3回以上　　）

**巧緻障害**なし　／　あり

　　　　　　　　　　　はし：　可　／　ぎこちない　／　大きなもののみつかめる　／　不可

　　　　　　　　　　　書字：　可　／　ぎこちない　／　　　何とかできる　　　／　不可

　　　　　　　　　　ボタン：　可　／　袖のボタン可　／　大きなボタン可　　　／　不可

**歩行**　正常　／　ぎこちないが速歩可能　／　平地では支持不要も階段降りるときに支持必要

　　　平地では支持不要も階段昇降に支持必要　／　平地では支持不要も不安定

　　　平地でも支持が必要　／　立位は可能も歩行不可　／　立位・歩行不可

**下肢関節痛**　　なし　／　あり　：　股関節　、膝関節、足関節

**下肢関節手術**なし　／　あり　：　股関節　、膝関節、足関節

　　　　　　　　　　　　　　　　　内容（部位、方法、病院など）

**問診からみて頚椎疾患の可能性は？**

　　　　　　　なし　／　あり　（　頚椎症　／　頚髄症　／　神経根症　）

**母指から中指（環指）のしびれ**　　　　　　　なし　／　右　／　左　／　両手

**上記しびれによる夜間覚醒**なし　／　あり

**上記のしびれは手を振ると軽快する**なし　／　あり

**問診からみて手根管症候群の可能性は？**なし　／　あり

**小指（環指）のしびれ**なし　／　右　／　左　／　両手

**上記のしびれは肘を曲げて出現する？**なし　／　あり

**問診からみて肘部管症候群の可能性は？**なし　／　あり

**整形外科受診／治療歴（内容と病院名を記載すること）：**
