# Supplementary material for: Epidemiological study of cervical cord compression and its clinical symptoms in community-dwelling residents
Source: PLoS One. 2021 Aug 27;16(8):e0256732. doi: 10.1371/journal.pone.0256732 (PMC8396744; doi:10.1371/journal.pone.0256732)
Supplement: Attachment — Submitted filename: 20210429 ResponseToReviewers.docx [file pone.0256732.s004.docx]

Response to journal requirements

1. Please ensure that your manuscript meets PLOS ONE's style requirements, including those for file naming. The PLOS ONE style templates can be found at

https://journals.plos.org/plosone/s/file?id=wjVg/PLOSOne_formatting_sample_main_body.pdf and

https://journals.plos.org/plosone/s/file?id=ba62/PLOSOne_formatting_sample_title_authors_affiliations.pdf

Response: Thank you very much for your comments. We have made the necessary changes.

2. Please note that PLOS does not permit references to “data not shown.” Authors should provide the relevant data within the manuscript, the Supporting Information files, or in a public repository. If the data are not a core part of the research study being presented, we ask that authors remove any references to these data.

Response: We have deleted “data not shown”.

*Assessment of the degree of cervical cord compression*

*P11, Line 181. In the measurement of AP diameter, intra-observer reliability was ρ = 0.73, and inter-observer reliability was ρ = 0.82. [~~data not shown~~].*

3. In your Methods section, please provide additional information about the participant recruitment method and the demographic details of your participants. Please ensure you have provided sufficient details to replicate the analyses such as:

a) the recruitment date range (month and year),

b) a description of any inclusion/exclusion criteria that were applied to participant recruitment,

c) a statement as to whether your sample can be considered representative of a larger population,

d) a description of how participants were recruited, and

e) descriptions of where the research took place.

Response: We have provided the information requested.

a) This study was performed in March, August, and November of 2005.

Materials and Methods

*P4, line 77. In May, August, and November of 2005,* ・・・

b) In the annual checkup for 3236 applicants by local governments, subjects who underwent cervical MRI were recruited. When they were recruited, those who underwent cervical spinal cord surgery were excluded. After a medical interview, neurological examination, and MRI, subjects with visual impairment, dementia, brain surgery, fracture of the lower extremities, and poor quality MRI were excluded.

Materials and Methods

*P5, Line 83.* *When they were recruited, those who underwent cervical spinal cord surgery were excluded.*

*P5, Line 86. After a medical interview, neurological examination, and MRI, subjects with visual impairment, dementia, brain surgery, fracture of the lower extremities, and poor quality MRI were excluded.*

*.*

c) The number of subjects varied by age group. Therefore, the following was added regarding generalizability.

Discussion

*P18, line 286.・・・there was not representative of the actual age demographics in these places, especially concerning the population of people under the age of 40 years. It is necessary to consider whether the results of this study can be applied to young people.*

d) e) In the annual checkup of 3236 applicants, 582 people provided written, informed consent to undergo MRI, medical interview, and physical examination as a cervical spine medical examination by one board-certified spine surgeon (KO) at Tadami Town, Ina Village, and Tateiwa Village in Fukushima Prefecture. AP diameters of the cervical spinal cord were then measured using a workstation at Fukushima Medical University (Fukushima City, Fukushima Prefecture).

Materials and Methods

*P4, line 77.*

*・・・in the annual checkups conducted by local governments for 3236* *applicants・・・**, 582 people provided written, informed consent to undergo MRI, medical interviews, and physical examinations as a cervical spine medical examination by one board-certified spine surgeon (KO) in each place.*

*P11, line 163．* *All images were measured using a workstation (ZioCube, Mita, Minato-ku, Tokyo, Japan) at Fukushima Medical University (Fukushima City, Fukushima Prefecture) by one orthopedic surgeon (TH) who was blinded to the clinical information.*

4. Please provide a sample size and power calculation in the Methods, or discuss the reasons for not performing one before study initiation.

Response: We did not consider the sample size and do a power calculation before starting this study because we could not assume the number of persons who underwent MRI. A total of 582 subjects in this study were recruited from among those who underwent the annual checkup (3236 applicants). The age and sex distributions were approximately similar in the two groups (Table 1). Therefore, it was possible to think that the subjects were representative of the 3236 applicants. However, there is not representative of the actual age demographics in Tadami Town, Ina Village, and Tateiwa Village, especially concerning the population of people under the age of 40 years. It is necessary to consider whether the results of this study can be applied to young people. This point is described as one of the limitations.

Discussion

*P18, line 284．Fifth, we did not consider the sample size and do a power calculation before starting this study. Because we could not assume the number of persons who underwent MRI. As a result, there was not representative of the actual age demographics in these places, especially concerning the population of people under the age of 40 years. It is necessary to consider whether the results of this study can be applied to young people.*

5. Please include additional information regarding the survey or questionnaire used in the study and ensure that you have provided sufficient details that others could replicate the analyses. For instance, if you developed a questionnaire as part of this study and it is not under a copyright more restrictive than CC-BY, please include a copy, in both the original language and English, as Supporting Information.

Response: In this study, subjective symptoms were investigated based on an interview sheet. We have provided the interview sheet in both the original language and English.

6.Thank you for stating the following in the Source of funding Section of your manuscript:

"study was supported by a grant from the Fukushima Society for the Promotion of Medicine and a grant from the Fukushima Prefectural Hospitals. These
grants were mainly used for the cost of the MRI, research assistance, and printing and
posting the questionnaire."

We note that you have provided funding information that is not currently declared in your Funding Statement. However, funding information should not appear in the Acknowledgments section or other areas of your manuscript. We will only publish funding information present in the Funding Statement section of the online submission form.

Please remove any funding-related text from the manuscript and let us know how you would like to update your Funding Statement. Currently, your Funding Statement reads as follows:

 "unfunded studies"

 Please include your amended statements within your cover letter; we will change the online submission form on your behalf.

Response: We have removed funding-related text. We have also corrected the funding statement according to your comments.

7. Please amend the manuscript submission data (via Edit Submission) to include author Koji Otani, MD, DMSc, Miho Sekiguchi, MD, PhD, Shin-ichi Kikuchi, MD, PhD, and Shin-ichi Konno.

Response: We have amended the manuscript submission data

8. Your ethics statement should only appear in the Methods section of your manuscript. If your ethics statement is written in any section besides the Methods, please move it to the Methods section and delete it from any other section. Please ensure that your ethics statement is included in your manuscript, as the ethics statement entered into the online submission form will not be published alongside your manuscript.

Response: The ethics statement has been moved to the Methods suggestion, as required.

Material and Methods

*P4, line 74.* *This study was approved by the ethics committee of our university (No. 1880).*

9. Please include your tables as part of your main manuscript and remove the individual files. Please note that supplementary tables (should remain/ be uploaded) as separate "supporting information" files.

Response: We have made the necessary changes.

Reviewer #1: The authors have submitted what appears to be figures and graphs but no actual manuscript body. It is impossible to evaluate the quality of data without this. Hopefully this material will be provided at some point.

Response: We have put the figures and graphs in the manuscript body.

Reviewer #2

1) It is not clear how novel this study is. Many of the references are somewhat dated. The introduction refers to "numerous studies" and that the "relationship remains unclear". This doesn't really demonstrate why this study is any different than any of the other "numerous studies" besides adding incrementally to the literature.

Response: Thank you very much for your comments. As you indicate, it is unclear in the manuscript why this study is any different than other studies. In the literature, it was not clear how much cervical cord pressure (degree of compression of the spinal cord) resulted in physical symptoms. Therefore, we tried to quantify the degree of compression and examined its relationship to symptoms. We believe this is clinically important and valuable.

Introduction

*P4, line 65*

*On the other hand, it is known that there are asymptomatic cervical cord compressions of the image. However, the initial symptom by the cervical cord compression is not clear. In the first place, it is not clear how much cervical cord compression causes clinical symptoms.*

2) While the English and grammar are not terrible, there are areas where the grammar and use of non-scientific language could be improved.

Response: This manuscript was checked by a professional native English-speaking medical editor associated with Forte Science Communications (Tokyo, Japan) again. We have included their certificate of English-language editing.

3) It wasn't clear to me whether the examining surgeon was blinded to the MRI results at the time of examination.

Response: Subjective symptoms and objective findings were evaluated by KO before MRI. On the other hand, AP diameter was measured by TH who was blinded to the information about subjective symptoms and objective findings. Finally, these data were collated when they were analyzed.

Material and Methods

*P4, line80．・・・582 people provided written, informed consent to undergo MRI, medical interviews, and physical examinations as a cervical spine medical examination by one board-certified spine surgeon (KO) in each place.*

*P11, line 163．* *All images were measured using a workstation (ZioCube, Mita, Minato-ku, Tokyo, Japan) at Fukushima Medical University (Fukushima City, Fukushima Prefecture) by one orthopedic surgeon (TH) who was blinded to the clinical information.*

4) There is a missed opportunity here to also examine cumulative compression. The data are all there, the statistics just need to be run.

Response: The purpose of this study was to clarify the relationship between the magnitude and the clinical symptoms of single-level cord compression at the maximal pressure part of the cervical spine. We would like to investigate cumulative compression in our next study.

5) For the AP diameter measurement, it is not clear how many radiologists were utilized to obtain the intra and inter-observer reliability. Then this was deemed "acceptable" and only one radiologist's measurements were utilized. It is unclear whether this reliability was pre-determined or was determined using the data for this study. If more than one radiologist read for this study, why not average their readings for more accuracy and input? More information could be provided here.

Response: Inter-observer and intra-observer reliabilities were calculated before the analysis. 30 subjects were randomly chosen. It was judged that the results for intra-observer reliability (ρ = 0.73) and inter-observer reliability (ρ = 0.82) were acceptable. Therefore the measurements obtained by TH were analyzed in this study. This is because if one doctor's measurements are statistically reliable, they are more efficient than the average of multiple doctors' measurements, we considered.

Material and Methods

*P11, line 176.* *Intra-observer and inter-observer reliabilities were calculated before the study results were analyzed. To evaluate intra-observer reliability, 30 MRIs of the cervical spine were randomly selected, and 180 AP diameters of the spinal cord (from C2-3 to C7-T1 of each) were measured three times by one observer (TH) every two weeks. Furthermore, to evaluate inter-observer reliability, other 30 MRIs were measured by two other orthopedic surgeons. In the measurement of AP diameter, intra-observer reliability was ρ = 0.73, and inter-observer reliability was ρ = 0.82. The intra-observer and inter-observer reliabilities were considered acceptable. Finally, all measurements were performed by TH and these measurements were adopted in this study.*
